# Frequency of *BRCA1* and *BRCA2* mutations in ovarian cancer patients in South-East Poland

**DOI:** 10.1186/s13053-022-00219-z

**Published:** 2022-04-05

**Authors:** Andrzej Jasiewicz, Helena Rudnicka, Wojciech Kluźniak, Wojciech Gronwald, Tomasz Kluz, Cezary Cybulski, Anna Jakubowska, Jan Lubiński, Jacek Gronwald

**Affiliations:** 1 Laboratory of Clinical Genetics, Molecular Biology of Cancer and Translational Research, Faculty of Medicine, Rzeszow University, 1a Warzywna St, 35-310 Rzeszów, Poland; 2 Genetic Counseling Center, Subcarpatian Oncological Hospital, 18 Bielawskiego St, 36-200 Brzozów, Poland; 3 Department of Genetics and Pathology, International Hereditary Cancer Center, Pomeranian Medical University, 1 Unit Lubelskiej St, 71-252 Szczecin, Poland; 4 Department of Obstetrics and Gynecology, Faculty of Medicine, Fryderyk Chopin University Hospital, Rzeszow University, No 1, 2 Szopena St, 35-055 Rzeszów, Poland

**Keywords:** *BRCA1* and *BRCA2* mutation, Ovarian cancer, Poland

## Abstract

**Background:**

Mutations in BRCA1 and BRCA2 genes are well-established risk factors of breast and ovarian cancer. In our former study, we observed that approximately 6% of unselected ovarian cancer patients in the region of Podkarpacie (South-East Poland) carry BRCA1 causative founder variants, which is significantly lower than in other regions of Poland. Therefore, it is deeply justified to do research based on the sequencing of whole BRCA1 and BRCA2 genes.

**Methods:**

We examined 158 consecutive unselected cases of ovarian cancer patients from the region of Podkarpacie. We performed *BRCA1* and *BRCA2* genes Next-Generation Sequencing study in all cases.

**Results:**

Altogether, in 18 of 158 (11.4%) ovarian cancer patients with *BRCA1* or *BRCA2* pathogenic mutations were found. *BRCA1* pathogenic variants were detected in 11 of the 158 (7.0%) ovarian cancer cases. 10 of 11 (91%) detected *BRCA1* mutations were founder mutations, detectable with the standard test used in Poland. *BRCA2* pathogenic variants were found in 7 of the 158 (4.4%) cases. No *BRCA2* pathogenic variants were founder mutations. The median age of patients at the diagnosis of the 18 hereditary ovarian cancers was 57.5 years.

**Conclusions:**

The frequency of *BRCA1* or *BRCA2* gene mutation carriers among patients with ovarian cancer from the Podkarpacie region is comparable to other regions of Poland. However, a significantly higher percentage of *BRCA2* gene mutations was observed, that were not detectable with a standard test for detection of founder mutations. Diagnostics based only on testing the *BRCA1/2* Polish founder mutations is characterized by relatively low sensitivity in the case of ovarian cancer patients from South-East Poland and should be supplemented by NGS study, in particular of the *BRCA2* gene.

## Introduction

The incidence of ovarian cancer is approximately 3,600 cases annually in Poland [1]. It has been shown that ovarian cancer patients from Poland are characterized by a high proportion of a limited number of recurrent mutations in the BRCA1 gene [2–7]. In 2003, Menkiszak et al. observed that 13.5% of ovarian cancer patients in the West Pomerania region carry one of these three common founder mutations in *BRCA1* (c.5266dupC, c.181T > G, and c.4035delA) gene [8]. A high proportion of carriers with a limited number of recurrent mutations have an impact on test costs reduction and testing effectiveness. Since 2003 some regional differences in *BRCA1* and *BRCA2* mutation frequency and spectrum have been reported [2–8]. In our latest study, conducted among consecutive patients with ovarian cancer from the Podkarpacie region (South-Eastern Poland), we observed only 6.3% *BRCA1* or *BRCA2* founder mutation carriers, and a slightly different spectrum of these mutations than in other regions of Poland [9].

The aim of this study was to define the prevalence and spectrum of *BRCA1* and *BRCA2* mutations in unselected ovarian cancer patients from the Region of Podkarpacie with the use of NGS, and establish an optimal algorithm for genetic testing of women diagnosed with ovarian cancer from this region.

## Methods

Ovarian cancer cases were identified from patients treated at a clinical base in the Department of Obstetrics and Gynecology of Fryderyk Chopin University Hospital No 1 in Rzeszow, Poland between January 2013 and January 2017. All patients were inhabitants of the South-East region of Poland. The study group consisted of 158 consecutive, newly diagnosed cases of ovarian cancer after surgical treatment, unselected for age or family history. The mean age of diagnosis was 58.5 years (range 22–84 years). The reference pathologist reviewed a representative slide from each cancer to confirm the diagnosis. 20% of patients were diagnosed in the I and II clinical stages according to FIGO, and 80% in III and IV. 14.8% of ovarian cancers showed pathological grading G1, 11.1 - G2, and 74.1 – G3. A cancer family history was obtained during an appointment with a clinical geneticist.

DNA was isolated from 5 to 10 ml of blood. We performed *BRCA1* and *BRCA2* genes Next-Generation Sequencing (NGS) study in all cases.

### NGS

*BRCA1* and *BRCA2* genes were screened using the SureMASTR BRCA Screen Kit from Agilent Technologies. The SureMASTR BRCA Screen (Agilent Technologies) analyses the full coding regions of these genes. In brief, 50ng (2.5ng/µl) of genomic DNA (isolated from a peripheral blood sample from each patient) was used to amplify the target genes in a single-tube multiplex reaction. The obtained amplicon libraries were purified and diluted before single-tube universal PCR reaction to tag all amplicons with specific p5 and p7 adaptors. Each tagged amplicon library was purified to remove small residual DNA fragments, and the DNA library concentration was quantified using a Pre-proof High Sensitivity Qubit quantification kit (Life Technologies). Equimolar quantities of the individually tagged amplicon libraries were pooled, and the final sequencing library was normalized to a concentration of 4nM. Sequencing was performed on a MiniSeq platform (Illumina) using the MiniSeq Mid Output Kit, 2 × 150 cycles, to obtain reads for both strands. All detected pathogenic mutations and variants of unknown significance (VUS) were validated using Sanger sequencing. The conventional Sanger sequencing was performed with the use of BigDye terminator sequencing kit v3.1 (Life Technologies) on the ABI Prism 3130 genetic analyzer (Life Technologies) according to the manufacturer’s protocol.

### Bioinformatics analysis

Bioinformatics analysis was performed using the software MASTR Reporter (Agilent Technologies). This analysis included read alignment to the human reference genome (Genome Reference Consortium GRCh37), variant calling, and visualization of the sequence reads. Variants above 40X coverage depth and with a minimum variant allele frequency of 5% for germline analysis are displayed in the software. Further filtering was applied to select germline variants which had a minimum of 100X coverage depth. Each sample passed quality control analysis.

### Variant filtering and classification

The clinical significance and the implications of variants were identified based on the annotations in public archives: ClinVar, Breast Cancer Information Core, Leiden Open Variation Database Online Mendelian Inheritance in Man (OMIM), Human Genome Variation Society (HGVS), and VarSome. Germline variants were classified according to the ACMG Standards and Guidelines for the Interpretation of Sequence Variants [10].

## Results

A *BRCA1* or *BRCA2* causative variant was found in 18 of 158 (11.4%) unselected ovarian cancer cases. A *BRCA1* mutation was detected in 11 (7.0%) patients. The c.5266dupC mutation was the most common, it was diagnosed in six patients, followed by the c.181T > G mutation observed in three patients, and the c.676delT mutation in one patient. All carriers of these 3 mutations were diagnosed previously with the test based on detection of founder pathogenic variants characteristic for the Polish population [9]. In addition, we diagnosed one *BRCA1* c.5346G > A mutation carrier (Table 1) with NGS only. A *BRCA2* gene mutation was diagnosed in 7 (4.4%) unselected ovarian cancer cases. None of these *BRCA2* mutations was a recurrent mutation characteristic for the Polish population. We also found in 3 patients, variants of unknown significance (VUS), all in the *BRCA2* gene (c.9302T > C, c.2063 A > G, and c.8527 A > G). The median age of diagnosis of the 18 hereditary ovarian cancers was 57.50 years (range 41–82 years), compared with a median age of diagnosis of 58.77 years (range 22–84 years) for the 140 cases without a mutation. However, the median age of diagnosis in *BRCA1* carriers was lower than *BRCA2* carriers − 55.8 vs. 60.1 years, respectively. A *BRCA1* mutation was found in 3, and *BRCA2* in 1 of 31 (together − 12.9%) women diagnosed with ovarian cancer at or under the age of 50 compared to 8 *BRCA1* and 6 *BRCA2* carriers of 127 (together − 11.0%) women diagnosed at a later age. Among the 18 women with ovarian cancer and a *BRCA1* or *BRCA2* mutation, ten reported a first- or second-degree relative with breast or ovarian cancer (55.5%), and there was only a slight difference between *BRCA1* and *BRCA2* carriers (54.5% vs. 57.1%, respectively). A mutation was present in 25.6% (10/39) of ovarian cancer patients with a positive family history and in 6.7% (8/119) of women with a negative family history. A significant family history, defined as a presence of first- or second-degree relative with breast or ovarian cancer, was observed in 24.7% (39/158) of patients with ovarian cancer (Table 2).

**Table 1:** The frequency of founder mutations in unselected series of 158 ovarian cancer patients.

*BRCA1* mutation	N	%
c.5266dupC	6	3.8
c.181T > G	3	1.9
c.676delT	1	0.6
c.5346G > A	1	0.6
*BRCA2* mutation		
c.7007G > A	1	0.6
c.3975_3978dupTGCT	1	0.6
c.5238dupT	1	0.6
c.1384G > T	1	0.6
c.7615 C > T	1	0.6
c.4544delA	1	0.6
c.6267_6269delGCinsC	1	0.6
Together	18	11.4

**Table 2:** Prevalence of *BRCA1*and *BRCA2* mutations in ovarian cancer patients, by age of onset and family history.

	Number of cases	Number with a mutation			Proportion with a mutation (%)		
		*BRCA1*	*BRCA2*	*BRCA1/2 *together	*BRCA1*	*BRCA2*	*BRCA1/2 *together
Age group							
≤ 50	31	3	1	4	9.7	3.2	12.9
> 50	127	8	6	14	6.3	4.7	11.0
Family history							
Positive	39	6	4	10	15.4	10.2	26.6
Negative	119	5	3	8	4.2	2.5	6.7
All cases together	158	11	7	18	7.0	4.4	11.4

## Discussion

The region of Podkarpacie is located in the South-East corner of Poland, bordering Ukraine and Slovakia. In our previous study, we identified 10 of 158 (6.3%) of unselected cases of ovarian cancer from this region carried one of 13 founder mutations in the *BRCA1* or *BRCA2* genes [9]. This is less than in other regions of Poland where the frequency of *BRCA1* causative founder variants was observed in about 10-13.5% of ovarian cancer patients [2–8]. In this study, we performed the NGS study of *BRCA1/2* genes in the same group of 158 women affected with ovarian cancer and diagnosed 18 (11.4%) *BRCA1/2* mutation carriers. The frequency of individual *BRCA1/2* mutations observed in ovarian cancer patients is shown in Table 1. All ten founder mutations were confirmed with our observations performed with a genetic test based on the *BRCA1/2* founder mutations characteristic for the Polish population [9]. In addition, we diagnosed 1 carrier of *BRCA1* and 7 carriers of the *BRCA2* gene. These 8 mutations were detectable by whole sequencing only. Like in other regions of Poland, the most frequent mutation was the *BRCA1* c.5266dupC mutation observed in 30% (6/18) of all carriers and the *BRCA1* c.181T > G mutation found in 15% (3/18). Other 9 *BRCA1/2* mutations were observed in single patients and are rare in the Polish population. In contrary to our former observation the frequency of *BRCA1/2* mutation carriers in the group of ovarian cancer patients is only slightly lower than in other regions of Poland. However, we observed a significantly lower frequency of founder mutations, in particular, *BRCA1* c.5266dupC and to a lesser extent *BRCA1* c.181T > G. This phenomenon can be caused in general by the lower frequency of these mutations in South-East Poland. However, it should be noted that in this region the extensive genetic testing of *BRCA1/2* genes has been carried out in patients with ovarian and breast cancer, as well as, in healthy patients since the year 2000. The testing focused mainly on the detection of founder mutations. As a result, several hundred families with *BRCA1* founder mutations have been diagnosed so far and several hundred prophylactic adnexectomies have been performed. It should be taken into account that these *BRCA1* founder mutations carriers were thus protected against ovarian cancer, and therefore, we observe their lower representation among BRCA1/2 mutation carriers who have now developed ovarian cancer.

The mean age at diagnosis in the 11 cases with *BRCA1* mutation was 55.8 years, and of the 7 patients with *BRCA2* mutation was 60.1 years. In both groups, the mean age at diagnosis was slightly higher than the observed in *BRCA1/2* carriers from other regions of Poland [6–8]. Possibly there are lifestyle/environmental factors which may influence the later age of diagnosis in *BRCA1/2* carriers. However, for non-carriers, the mean age at diagnosis was similar in the region of Podkarpacie and the rest of Poland (58.77 vs. 56.2–62.3 years) [6–8].

We observed strong family history in 10 of 18 (55.5%) mutation carriers, which is slightly more frequent than in other regions [6–8]. This applies to families with mutations in the *BRCA1* as well as the *BRCA2* gene. It can be explained by the relatively larger number of family members in an average family, in this region. However, the frequency of *BRCA1/2* mutation carriers with negative family history is so high (44.5%), both in groups with recurrent founder mutations as well as with non-founder mutations, that we cannot recommend limiting performing the *BRCA1* and *BRCA2* gene testing based on NGS to cases with a burdened family history only. Also, the ovarian cancer age of onset is not a factor facilitating the qualification for this study. Taking into account our observations, it should be stated that performing a test based on the detection of Polish founder mutations in ovarian cancer patients from the Podkarpacie region is associated with relatively low sensitivity (55.5%). In turn, performing the NGS test in all subsequent patients with ovarian cancer is associated with a significant increase in costs. Application of NGS tests only in familial cases is associated also with low sensitivity of 55.5%. One of the compromise solutions would be to perform a standard genetic test based on the detection of founder mutations in all patients, and then if no mutation is detected, perform NGS in cases with a family history. With this algorithm of procedure, the sensitivity of detecting the *BRCA1/2* gene mutation in ovarian cancer patients would increase to 77.8%, at a relatively low cost.

However, taking into account the current diagnostic standards of patients with ovarian cancer in the context of determining the optimal treatment and qualifying patients for treatment with PARP inhibitors, the *BRCA1/2* gene is routinely tested using the NGS method in DNA extracted from tumour cells. It seems, therefore, that the most justifiable algorithm for detecting a germinal mutation in these patients is to start testing *BRCA1/2* genes using the NGS method in DNA extracted from tumour cells. Then, if a mutation is found, the test should be performed in the patient’s peripheral blood to verify whether it is a germline or somatic mutation. If germline mutation is confirmed, the study should be extended to other relatives. However, if such a protocol is used, it should be taken into account that up to nearly 10% of cancer patients with negative NGS results performed in DNA isolated from neoplastic tissue cells, in fact, may carry germline mutation [11]. Lincoln et al. [11] indicated that major reasons for the mutations omissions include: (i) somatic variant interpretation guidelines differ from germline variant interpretation guidelines [12, 13]; (ii) high-quality germline tests can detect a broad spectrum of pathogenic variant types but present technical challenges particularly when analyzing archival specimen types often encountered in oncology e.g., paraffin-embedded formalin-fixed tissues [14–17], (iii) tumor tests may not include all genes of potential germline relevance in any given patient. Since the material for somatic mutation tests is mainly derived from tissues embedded in paraffin blocks, a significant percentage of the isolated DNA is of poor quality and unsuitable for NGS tests. It should be noted that patients with inconclusive tumor tests may also be carriers of germline mutations [18]. Therefore, the results of NGS tests carried out in somatic tissues should be interpreted with special care.

## Conclusions

Approximately 11% of unselected ovarian cancer patients in the region of Podkarpacie carry a *BRCA1* or *BRCA2* causative variants. We found a significantly higher percentage of *BRCA2* gene mutations, which are not detectable with a standard test for founder mutations. Diagnostics based only on testing the *BRCA1/2* Polish founder mutations is characterized by relatively low sensitivity in the case of ovarian cancer patients from South-East Poland and should be supplemented by NGS study, in particular of the *BRCA2* gene.

## Data Availability

The datasets used and/or analyzed during the current study are available from the corresponding author on reasonable request.

## Abbreviations

PCR: Polymerase chain reaction
NGS: Next-Generation Sequencing
FIGO: International Federation of Gynaecology and Obstetrics
